# Plasma Betatrophin Levels and Carotid Atherosclerosis

**DOI:** 10.1155/2019/4214650

**Published:** 2019-10-17

**Authors:** Hanako Niki, Yoshimi Kishimoto, Emi Saita, Reiko Ohmori, Kazuo Kondo, Yukihiko Momiyama

**Affiliations:** ^1^Department of Cardiology, National Hospital Organization Tokyo Medical Center, Tokyo, Japan; ^2^Endowed Research Department “Food for Health”, Ochanomizu University, Tokyo, Japan; ^3^Faculty of Regional Design, Utsunomiya University, Tochigi, Japan; ^4^Institute of Life Innovation Studies, Toyo University, Gunma, Japan

## Abstract

**Aims:**

Betatrophin is a recently identified circulating adipokine that may affect lipid and glucose metabolism. However, the association between plasma betatrophin levels and carotid atherosclerosis has not been elucidated.

**Methods:**

We investigated plasma betatrophin levels in 153 subjects undergoing carotid ultrasonography. The severity of plaque was evaluated as plaque score.

**Results:**

Of the 153 subjects, plaque was found in 63 (41%). Plasma betatrophin levels were higher in 63 subjects with plaque than in 90 without plaque (median 906 vs. 729 pg/mL, *P* < 0.025). A stepwise increase in betatrophin levels was found depending on the plaque score: 729 pg/mL in score = 0 (*n* = 90), 802 pg/mL in score = 1 (*n* = 31), and 978 pg/mL in score ≥ 2 (*n* = 32) (*P* < 0.01). In particular, betatrophin levels in subjects with score ≥ 2 were higher than in those with score = 0 (*P* < 0.05). Moreover, betatrophin levels correlated with plaque score (*r* = 0.23, *P* < 0.01), but no significant correlation was found between betatrophin levels and triglyceride or HbA1c levels. The percentage of subjects with betatrophin > 800 pg/mL was higher in subjects with plaque than in those without plaque (65% vs. 44%) and was highest in score ≥ 2 (78%) (*P* < 0.005). In the multivariate analysis, betatrophin level was not a significant factor for the presence of plaque but was a significant factor for plaque score ≥ 2, independent of atherosclerotic risk factors. The odds ratio for score ≥ 2 was 4.9 (95% CI = 1.9-12.8) for betatrophin > 800 pg/mL.

**Conclusions:**

Plasma betatrophin levels were found to be high in subjects with carotid plaque and to be associated with the severity of plaque. Betatrophin may play a role in the progression of carotid atherosclerosis.

## 1. Introduction

Betatrophin, also called angiopoietin-like protein 8 (ANGPTL8), is a recently identified circulatory adipokine, mainly secreted from the liver and adipose tissues, which may affect both glucose and lipid metabolism [1]. In animal models, one study reported betatrophin to induce pancreatic beta cell proliferation [2], but another study showed betatrophin not to affect beta cell expansion nor glucose metabolism [3]. Most studies reported blood betatrophin levels to be high in patients with diabetes mellitus (DM) [4, 5], while some studies found no difference between DM and non-DM patients [6]. In lipid metabolism, betatrophin was suggested to inhibit lipoprotein lipase activity and to increase blood triglyceride (TG) levels [7]. Betatrophin knockout mice were also reported to exhibit lower TG levels in the fed state, but not in the fasted state [1, 7]. Moreover, one low-frequency variant of the betatrophin gene (rs145464906) was reported to be associated with lower TG levels but not with lower glucose levels [8]. However, some studies reported blood betatrophin levels to correlate with TG levels [5, 9], but others showed no correlation [4, 6]. Therefore, the associations between blood betatrophin levels and DM or TG levels remain controversial.

Regarding the association between blood betatrophin levels and atherosclerosis, one study reported that serum betatrophin levels were higher in 22 diabetic patients with coronary artery disease (CAD) or stroke than in 101 without such disease [10]. However, no study reported blood betatrophin levels in patients with carotid plaque. Therefore, we investigated the association between plasma betatrophin levels and carotid atherosclerosis.

## 2. Methods

### 2.1. Study Patients

The data that support the findings of this study are available from the corresponding author on reasonable request. We investigated plasma betatrophin levels in 153 consecutive subjects (mean age 65 ± 10 years, range 41 to 86 years) who underwent carotid ultrasonography as well as ankle-brachial index test for medical checkup to evaluate atherosclerosis at Tokyo Medical Center. Of the 165 study subjects, 9 with a history of cerebral infarction, CAD, or peripheral artery disease (PAD) were excluded from this study. Since fibrates reduce serum triglyceride (TG) levels intensively, 3 subjects taking fibrate were excluded, and hypertriglyceridemia was defined as a TG level of >150 mg/dL. Hypercholesterolemia was defined as an LDL cholesterol level of >140 mg/dl or on drugs, and 28 (18%) subjects were taking statins. Hypertension was defined as blood pressure of ≥140/90 mmHg or on drugs, and 54 (35%) were taking antihypertensive drugs. DM (a fasting plasma glucose (FPG) level of ≥126 mg/dl or on treatment) was present in 10 (7%) subjects, and 15 (10%) were smokers (≥10 pack-years). Our study was approved by the institutional ethics committee of our hospital (approval no. R07-054/R16-012), and written informed consent was obtained from all study subjects.

### 2.2. Carotid Ultrasonography

Both the right and left carotid arteries were evaluated from the longitudinal and transverse views of the common (CCA), bifurcation, and internal carotid arteries (ICA) using a high-resolution B-mode ultrasonography (Aplio 400, Toshiba, Japan). Intima-media thickness (IMT) was measured in triplicate using a computer-assisted method by experienced sonographers blinded to the clinical and laboratory data. Mean IMT was defined as the average of the mean values from the distal 1 cm of the far walls of both right and left CCA. Plaque was defined as a focal wall thickening of ≥1.5 mm or ≥50% of the surrounding IMT [11]. The severity of plaque was evaluated as a plaque score which was calculated as the sum of points (range 0 to 12) of all 6 segments. In each segment, 1 point per plaque was allocated for the near and far walls of each segment (CCA, bifurcation, and ICA) of the right and left carotid arteries [12].

### 2.3. Measurements of Plasma Betatrophin and C-Reactive Protein (CRP) Levels

Overnight-fasting blood samples were taken on the day of medical checkup. Plasma was stored at –80°C. Plasma betatrophin levels were measured by an enzyme-linked immunosorbent assay (ELISA) with a commercially available kit (WUHAN EIAab Science; Catalog number E11644h, China) at Ochanomizu University according to the manufacturer's instructions. The intra- and interassay coefficients of variation were <8% and <10%, respectively. Plasma high-sensitivity C-reactive protein (hsCRP) levels were also measured by a BNII nephelometer (Dade Behring, Tokyo, Japan).

### 2.4. Statistical Analysis

Differences between 2 groups were evaluated by the unpaired *t*-test for parametric variables, by the Mann–Whitney *U* test for nonparametric variables, and by the chi-squared test for categorical variables. Differences among 3 groups were evaluated by an analysis of variance with Scheffe's test for parametric variables, by the Kruskal-Wallis test with the Steel-Dwass test for nonparametric variables, and by the chi-squared test for categorical variables. Since the distributions of measured betatrophin and hsCRP levels were considered to be highly skewed and to be nonparametric variables by the Shapiro-Wilk test, these results were presented as the median value. Moreover, correlations with betatrophin levels were evaluated by Spearman's rank correlation test. To determine the cut-off point of betatrophin levels for carotid plaque, a relative cumulative frequency distribution curve was created, and then, the optimum cut-off point was determined to be 800 pg/mL. A multiple logistic regression analysis was used to determine the independent association between betatrophin levels and plaque. Variables entered into a multiple logistic regression model were age, gender, hypertension, hypercholesterolemia, statin use, hypertriglyceridemia, DM, smoking, and betatrophin level (>800 pg/mL). The optimum cut-off point of age was also determined to be 65 years by a relative cumulative frequency distribution curve. A *P* value of <0.05 was considered to be statistically significant. Results are presented as the mean ± SD or the median value.

## 3. Results

**Figure 1:**
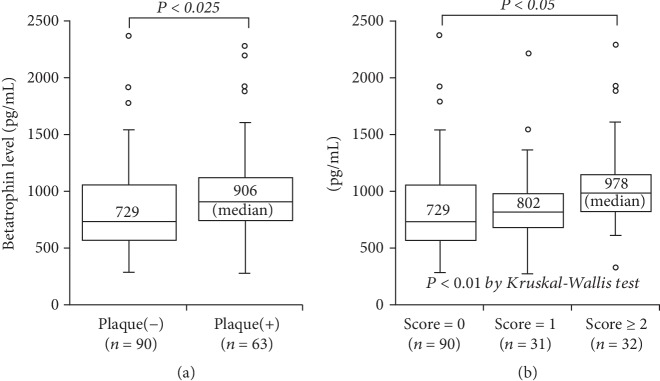
Betatrophin levels and carotid plaque or plaque score. Plasma betatrophin levels were significantly higher in subjects with plaque than in those without plaque (a) (*P* < 0.025). A stepwise increase in betatrophin levels was found depending on the plaque score: 729 pg/mL in score = 0, 802 pg/mL in score = 1, and 978 pg/mL in score ≥ 2 (*P* < 0.01). In particular, betatrophin levels in score ≥ 2 were higher than those in score = 0 (*P* < 0.05) (b).

Of the 153 study subjects, carotid plaque was found in 63 (41%), of whom 31 had a plaque score of 1, 21 had a score of 2, 8 had a score of 3, and 3 had a score of 4. Compared with 90 subjects without plaque, 63 subjects with plaque were older (69 ± 9 vs. 62 ± 10 years) and predominantly male (*P* < 0.025) (Table 1). Plasma hsCRP levels tended to be higher in subjects with plaque than in those without plaque (median 0.38 vs. 0.34 mg/L), but this difference did not reach statistical significance. However, plasma betatrophin levels were significantly higher in subjects with plaque than in those without plaque (median 906 vs. 729 pg/mL, *P* < 0.025) (Figure 1). A stepwise increase in betatrophin levels was found depending on the plaque score: 729 pg/mL in score = 0 (*n* = 90), 802 pg/mL in score = 1 (*n* = 31), and 978 pg/mL in score ≥ 2 (*n* = 32) (*P* < 0.01) (Figure 1). In particular, betatrophin levels in subjects with score ≥ 2 were higher than those in subjects with score = 0 (*P* < 0.05). Furthermore, betatrophin levels significantly correlated with the plaque score (*r* = 0.23) and the mean IMT (*r* = 0.22) (*P* < 0.01), but no significant correlation was found between betatrophin levels and TG, FPG, or HbA1c levels. The percentage of subjects with betatrophin level > 800 pg/mL was higher in subjects with plaque than in those without plaque (65% vs. 44%) and was highest in score ≥ 2 (78%) (*P* < 0.005) (Table 1). In the multivariate analysis, betatrophin level was not a significant factor for the presence of plaque, but it was a significant factor for the plaque score ≥ 2, independent of atherosclerotic risk factors including DM and TG level. The odds ratio for the score ≥ 2 was 4.9 (95% CI = 1.9-12.8) for betatrophin level > 800 pg/mL (Table 2).

## 4. Discussion

In the present study, plasma betatrophin levels were significantly higher in subjects with carotid plaque than in those without plaque, and they correlated with the severity of plaque, defined as plaque score. Betatrophin levels were found to be associated with carotid plaque, especially severe plaque (score ≥ 2), independent of DM and TG levels.

Betatrophin is suggested to affect both glucose and lipid, especially TG, metabolism [1]. However, its effects on DM or TG levels remain controversial [4–7, 9]. Although the number of our study subjects was small (*n* = 153), we found no correlation between plasma betatrophin and TG, FPG, or HbA1c levels. Any subjects taking fibrate were excluded from our study, but 28 (18%) of 153 subjects were taking statins which can reduce TG levels. Although some studies reported the correlation between FPG or HbA1c and betatrophin levels in diabetic patients [4, 13], only 10 (7%) subjects had DM in our study. These may be the reasons why plasma betatrophin levels did not correlate with TG, FPG, or HbA1c levels.

Regarding betatrophin levels and atherosclerosis, Maurer et al. [9] reported that betatrophin levels correlated with carotid IMT (*r* = 0.26) among 535 subjects, of whom 32 (6%) had DM. Our present study also found that plasma betatrophin levels significantly, but weakly, correlated with carotid IMT (*r* = 0.22). Notably, we demonstrated betatrophin levels to be higher in 63 subjects with carotid plaque than in 90 subjects without plaque and to correlate with the severity of plaque (the plaque score). Moreover, betatrophin levels were found to be associated with carotid plaque, especially severe plaque (score ≥ 2), independent of atherosclerotic risk factors including DM and TG level. Our findings thus suggest that betatrophin plays a role in the progression of carotid plaque. Recently, we investigated plasma betatrophin levels in 457 patients undergoing both coronary angiography and ankle-brachial index test [14]. We reported plasma betatrophin levels to be high in patients with CAD and those with PAD and to correlate with the severity of CAD and PAD. Betatrophin levels were a significant factor associated with CAD, especially 3-vessel disease, as well as PAD, independent of atherosclerotic risk factors. Thus, the results of both our previous [14] and present studies suggest that betatrophin plays a role in the progression of atherosclerosis, independent of glucose and TG metabolism, and that betatrophin levels may be one of the atherosclerotic risk factors.

Betatrophin (ANGPTL8) is recognized to be an atypical member of the ANGPTL protein family, because of the lack of a fibrinogen-like domain which is present in the typical ANGPTL protein family. However, betatrophin has some similar gene structure to ANGPTL3 [1]. In vivo, reduced ANGPTL3 expression reduced atherosclerosis [15]. Plasma ANGPTL3 levels was also reported to correlate with carotid IMT [16]. Hence, ANGPTL3 may have a promotive effect on atherosclerosis. Interestingly, betatrophin and ANGPTL3 were shown to cooperate in the regulation of TG levels [17, 18]. Betatrophin also promotes the ability of ANGPTL3 to bind and inhibit lipoprotein lipase (LPL) [19, 20]. Betatrophin is known to be mainly secreted from the liver and adipose tissues [1], but no study has reported any betatrophin mRNA expression or protein in atherosclerotic lesions. Moreover, one low-frequency variant of the betatrophin gene (rs145464906) was reported to be associated with lower TG levels [8], but there was no study showing any betatrophin gene variant associated with atherosclerotic disease. Therefore, the direct atherogenic effect of betatrophin and the mechanism how betatrophin affects atherosclerosis have not been clarified yet. Further studies are needed to elucidate atherogenic effect of betatrophin and to measure both betatrophin and ANGPTL3 levels in blood as well as LPL activity.

Our study have several limitations. One of the major limitations is a small number of study subjects (*n* = 153). Moreover, we did not measure ANGPTL3 levels and LPL activity. Finally, our study was cross-sectional in nature, and it could not establish causality, since it only showed some associations and proposed some hypotheses.

In conclusion, plasma betatrophin levels were found to be high in subjects with carotid plaque and to be associated with the severity of plaque. Betatrophin may thus play a role in the progression of carotid atherosclerosis.

## Figures and Tables

**Table 1:** Clinical characteristics of subjects with and without plaque.

	Plaque (-) (*n* = 90)	*P* value	Plaque (+) (*n* = 63)	Plaque score = 1 (*n* = 31)	Plaque score ≥ 2 (*n* = 32)
Age (years)	62 ± 10	*<0.001*	69 ± 9	68 ± 9	70 ± 8
Gender (male)	42 (47%)	*<0.025*	42 (67%)	20 (65%)	22 (69%)
Body mass index (kg/m^2^)	23.1 ± 3.4	*NS*	22.9 ± 3.9	22.8 ± 4.0	23.0 ± 4.0
Hypertension	43 (48%)	*NS*	35 (56%)	15 (48%)	20 (63%)
Systolic BP (mmHg)	124 ± 14	*NS*	128 ± 16	128 ± 15	128 ± 18
DM	4 (4%)	*NS*	6 (10%)	3 (10%)	3 (9%)
HbA1c (%)	5.8 ± 0.4	*NS*	5.9 ± 0.8	6.1 ± 1.0	5.8 ± 0.5
Smoking	10 (11%)	*NS*	5 (8%)	3 (10%)	2 (6%)
Hypercholesterolemia	38 (42%)	*NS*	26 (41%)	13 (42%)	13 (42%)
Statin	17 (19%)	*NS*	11 (17%)	6 (19%)	5 (16%)
LDL cholesterol (mg/dL)	127 ± 32	*NS*	124 ± 32	123 ± 31	124 ± 34
HDL cholesterol (mg/dL)	62 ± 17	*NS*	63 ± 15	65 ± 16	62 ± 14
Hypertriglyceridemia	19 (21%)	*NS*	14 (9%)	6 (19%)	3 (9%)
TG (mg/dL)	111 ± 103	*NS*	95 ± 53	96 ± 64	94 ± 39
hsCRP levels (mg/L)	0.34 [0.19, 0.68]	*NS*	0.38 [0.19, 0.83]	0.35 [0.14, 0.68]	0.38 [0.19, 0.90]
Betatrophin levels (pg/mL)	729 [569, 1046]	*<0.025*	906 [728, 1116]	802 [672, 976]	978 [819, 1139]
Betatrophin >800 pg/mL	40 (44%)	*<0.025*	41 (65%)	16 (52%)	25 (78%)
Carotid ultrasonography					
Mean IMT (mm)	0.77 ± 0.13	*<0.001*	0.87 ± 0.17	0.86 ± 0.18	0.88 ± 0.16
Plaque score	0.0 ± 0.0	*<0.001*	1.7 ± 0.9	1.0 ± 0.0	2.4 ± 0.7

Data represent the mean ± SD or the number (%) of subjects, except for hsCRP and betatrophin levels presented as the median value and interquartile range. SBP = systolic blood pressure; TG = triglyceride.

**Table 2:** Factors associated with carotid plaque.

	Odds ratio	95% CI	*P* value
Plaque (+)			
Age (>65 years)	2.55	1.30-5.01	*<0.01*
Male gender	2.21	1.11-4.38	*<0.025*
Plaque score ≥ 2			
Betatrophin level (>800 pg/mL)	4.91	1.88-12.82	*<0.002*

The dependent variables were the presence of plaque or the plaque score ≥ 2. The analysis included age (>65 years), gender, hypertension, hypercholesterolemia, statin use, hypertriglyceridemia (TG > 150 mg/dL), DM, smoking, and betatrophin level (>800 pg/mL).

## Data Availability

The data that support the findings of this study are available from the corresponding author on reasonable request. We investigated plasma betatrophin levels in 153 consecutive subjects (mean age 65 ± 10 years, range 41 to 86 years) who underwent carotid ultrasonography as well as ankle-brachial index test for medical checkup to evaluate atherosclerosis at Tokyo Medical Center.
